# The Origin and the Adaptive Function of Genetic Recombination in Sexual Reproduction

**DOI:** 10.3390/genes17070750

**Published:** 2026-06-29

**Authors:** Carol Bernstein, Harris Bernstein

**Affiliations:** Department of Cellular and Molecular Medicine, College of Medicine, University of Arizona, Tucson, AZ 85721, USA; bernstein79@zohomail.com

**Keywords:** DNA damage, DNA repair, sexual reproduction, recombination, breakage and exchange, synthesis-dependent strand annealing

## Abstract

Genetic recombination occurs in many organisms, from simple RNA viruses to mammals and plants with DNA genomes. In sexual reproduction, two parental genomes come together and undergo recombination, producing an offspring genome with a combination of information from the two parental genomes. Genome recombination that occurs during sexual reproduction can involve any one of several mechanisms, including copy-choice recombination as well as breakage and exchange. Across widely different organisms, recombination is generally promoted by factors that damage the genetic material. In organisms such as bacteriophage and *Paramecium*, it was experimentally demonstrated that recombinational repair during sexual reproduction can overcome otherwise deleterious or lethal damage. For many decades, it has been recognized that there are higher biological costs of sexual reproduction than for asexual reproduction. Theories assuming that genetic variation, due to recombination, is the main adaptive benefit of sexual reproduction have been widely accepted. Such a benefit was considered to compensate for the high cost of sexual reproduction. However, it has been difficult to find a strong, consistent benefit of variation. The repair of lethal damage, involving recombinational interactions of two different genomes, now appears to be the major selective factor underlying sexual reproduction in organisms both simple and complex.

## 1. Origin of Sexual Reproduction as Recombination Between Homologous RNA Genomes

Asexual reproduction is a process by which a single genome reproduces itself and gives rise to progeny with the same genetic sequence as the parent (a clone).

Sexual reproduction is a process in which two parental genomes participate in producing an offspring genome that has a combination of information from the two parental genomes.

Recombinational repair of nucleotide damage can occur via several mechanisms, including synthesis-dependent strand annealing (copy-choice) and breakage and exchange. Two homologous lengths of nucleotide chains (DNA or RNA) are paired, and a damaged segment or broken end of one homologue is replaced by copying sequence information from the second homologue. The strands are then rearranged, or broken and rearranged, to give rise to two intact homologous nucleotide chain segments without damage that carry genetic information from both original nucleotide chains.

As noted by Haynes and Kunz in 1988 [1], “DNA is composed of rather ordinary molecular subunits, and certainly is not endowed with any peculiar kind of physicochemical stability… Its very chemical vulgarity makes it prey to all the horrors and misfortune that might befall any such molecule in a warm aqueous medium… It is also subject to damages from highly reactive free radicals, peroxides, singlet oxygen, reducing agents, etc., as well as low but sometimes significant exposures to ionizing and ultraviolet radiations.” This also applies to RNA when it is employed as the genetic material. DNA and RNA genomes are both subject to damage, and damaged genomes require mechanisms to repair the damage.

By 1993, Ames [2] was able to estimate that in a human cell nucleus, there were about 10,000 new oxidative damages to human nuclear DNA per day. By 2011, Swenberg et al. [3] were able to show that the steady-state level of one of the types of oxidative DNA damage, 8-hydroxyguanine (which is 5% of the oxidative damage), was present as 2400 DNA-damaged molecules in each cell (after both incident damage and repair of damage had occurred). In 2013, Cadet and Wagner [4] showed that oxidative DNA damage included interstrand cross-links. Interstrand cross-links are repaired by a recombinational repair process [5].

These DNA damages were largely due to cellular oxidative metabolism in the mitochondria, which leaks superoxide (O_2_·^−^) into the cytoplasm [6]. In the cytoplasm, superoxide is converted into hydrogen peroxide (H_2_O_2_) and molecular oxygen [6]. H_2_O_2_ is small and not very reactive, and it can diffuse through the cytoplasm and through the nuclear membrane. In the nucleus, H_2_O_2_ can react with chromatin-associated iron to undergo the Fenton reaction, creating the strongly reactive oxygen species, the hydroxyl radical OH, which can cause oxidative damage to DNA [7].

Note that the mitochondria in eukaryotic cells are descendants of a once free-living oxidatively metabolizing alphaproteobacterial cell that, through a singular endosymbiotic event about 1.2 billion years ago, became encapsulated into an Asgard archaeal cell [8,9]. All eukaryotic organisms are descended from this singular event. The alphaproteobacterium, through selection during evolution, gave rise to mitochondria.

The age of the Earth is 4.54 billion years, and it appears that an early life form consisting of RNA molecules that could replicate [10] arose roughly a billion years later [11]. These life forms were thought to have arisen about 3.8 to 4.2 billion years ago [12,13]. This time period was called the RNA world.

A first requirement for an RNA life form is replication of its genome. For replication in the early RNA world, an RNA genome must have been able to form a ribozyme (an RNA molecule with catalytic activity) that could replicate RNA [14]. Thus, progeny RNA molecules could be produced. But, at the same time, there is a need to repair damage to the RNA genomes. Thus, genetic repair must have emerged soon after the first RNA genomes were formed.

Of course, in the RNA world, the RNA-damaging agents likely would have been different from the reactive oxygen species described by Haynes and Kunz [1] above. There was little atmospheric oxygen present in the RNA world. One estimate suggested that the atmosphere was likely composed of ammonia, methane, carbon monoxide, water vapor, nitrogen, hydrogen, and hydrogen sulfide (H_2_S) [15]. A more recent estimate indicated the atmosphere consisted of carbon dioxide, sulfur dioxide, water, and nitrogen gas [16]. The major damage to RNA replicators may have been due to UV light, since the Earth did not have a protective ozone layer at that time to block UV light from the sun [16]. But the point of Haynes and Kunz [1] is still valid, as there would have been damage to RNA replicators.

Genome repair in the RNA world was probably similar to the genome repair of single-stranded RNA viruses in the current day. This repair would operate by a copy-choice recombination model. This is where an RNA-dependent RNA polymerase (RdRp) (formed by a ribozyme) would replicate along one strand of an RNA polynucleotide genome and then encounter a damaged nucleotide that could not be copied. At this point, the RdRp would switch to a nearby polynucleotide RNA genome to continue replication. If the RdRp were to encounter another damaged nucleotide, the RdRp could switch back to the original polynucleotide RNA genome and continue copying there. As reviewed by Barr and Fearns [17], this type of copy-choice repair mechanism appears to occur in a number of extant single-stranded viruses and can be extremely efficient. This type of recombination avoids damage rather than directly repairing damage. In early RNA replicators, recombinational repair may have been central to the sexual reproduction process.

## 2. Genetic Recombination in RNA Viruses: An Adaptation for RNA Repair

**Figure 1:**
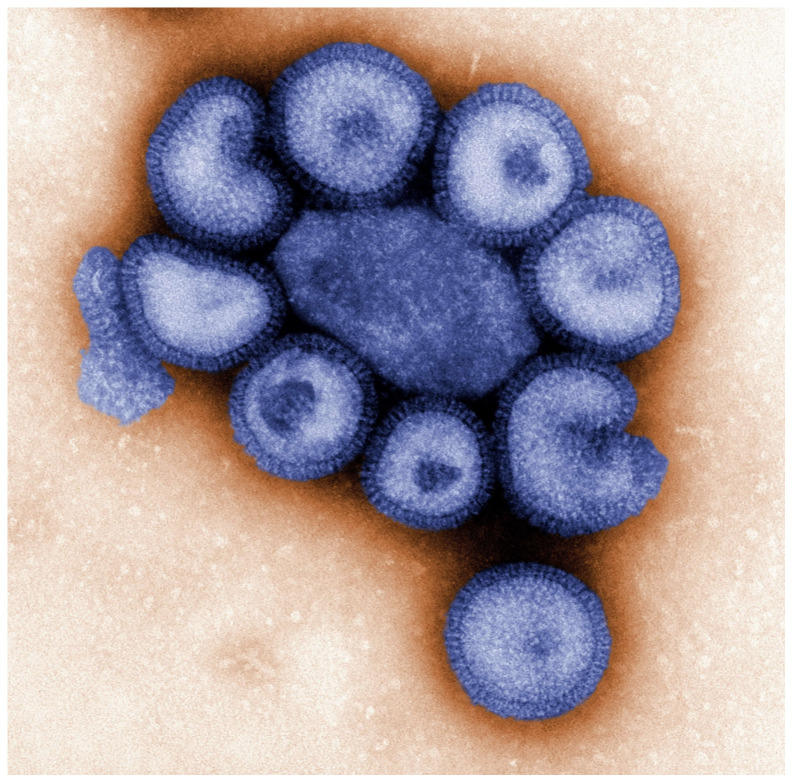
Influenza virus. An enveloped, segmented, negative-sense, single-stramded RNA virus. By Dr. F.A. Murphy CC0 license.

One single-strand virus for which copy-choice recombinational repair has been reported is the retrovirus “spleen necrosis virus” [18]. The authors stated, “when the viral RNA genomes are damaged, intermolecular transfer (copy choice strand switching) of minus strand DNA occurs.” Similarly, in polio virus, copy-choice recombinational repair occurs at breaks in the RNA genome [19]. Barr and Fearns [17] reviewed the evidence for copy-choice recombinational repair in six RNA viruses: bacteriophage MS2, mouse hepatitis virus, bacteriophage Qβ, Sindbis virus, cowpea chlorotic mottle virus, and bunyavirus. In all six viruses, copy-choice recombinational repair was carried out by the viral RNA-dependent RNA polymerase (RdRp), and the recombinational event occurred co-transcriptionally during copying of the RNA template. In the RNA viruses described here, this appears to be sexual reproduction accompanied by recombinational repair. Figure 1 is an electron micrographic image of an RNA virus.

## 3. Genetic Recombination in DNA Viruses: An Adaptation for DNA Repair

**Figure 2:**
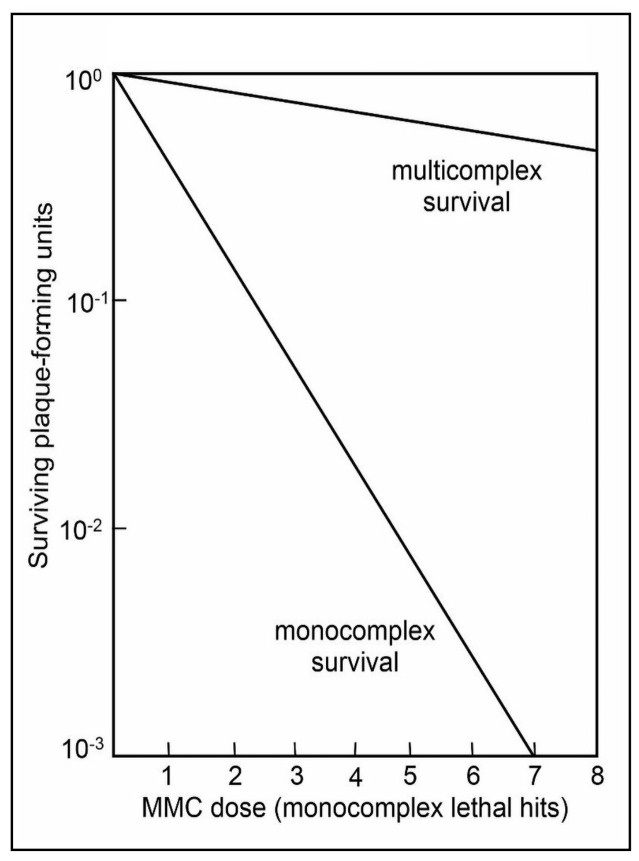
Multiplicity reactivation. Bacteriophage T4 were treated with increasing doses of mitomycin C (MMC), a DNA cross-linking agent. Multiplicity reactivation (greatly increased survival) is shown for bacteriophage plaque-forming ability of *Escherichia coli* cells multiply-infected with phage T4 compared to plaque-forming ability of singly-infected cells. By Chaya5260, CC by SA 4.0.

Bacteriophages (phages) are viruses that infect bacteria. Most bacteriophages use double-stranded DNA as their genetic material, though a minority of these viruses are RNA or single-stranded DNA phages [20]. A single phage can infect a host bacterium and reproduce asexually. A DNA phage with lethal damage to its genome may still be able to infect a host bacterial cell, but no viable progeny viruses will be produced. However, when two lethally damaged phage genomes enter in cells infected by multiple phage compared to cells infected by a single phage eachone bacterial cell by simultaneous infection, the two parental genomes may interact in such a way as to create functional progeny phages. This phenomenon is called multiplicity reactivation. Multiplicity reactivation can compensate for DNA damage caused by many different agents [21]. Figure 2 indicates the increase in survival of phages with DNA damage when infection occurs.

**Figure 3:**
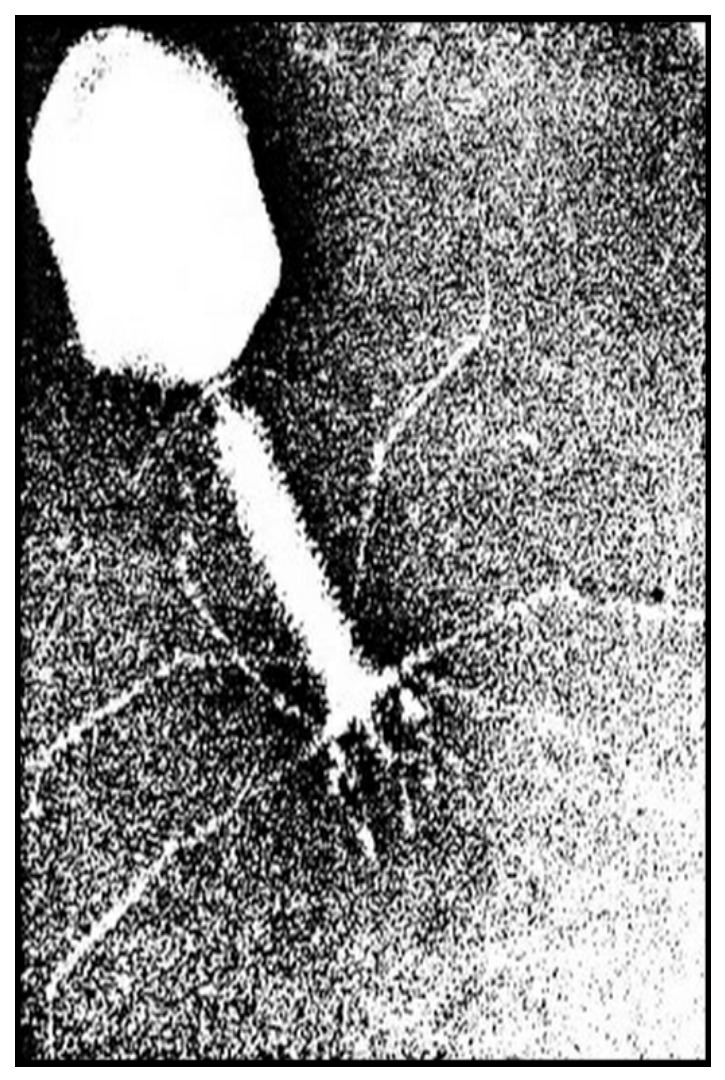
Phage T4 (electron micrograph). Creative Commons attribution 4.0 International by 7USSR7.

The model virus for multiplicity reactivation is phage T4 (see Figure 3), although multiplicity reactivation occurs in other phages as well [21]. Multiplicity reactivation in DNA phages occurs by a recombinational repair process that involves the interaction of two phage genomes in a single cell [21,22]. Multiplicity reactivation is therefore a form of sexual reproduction since it involves two homologous genomes from separate phages that come together in such a manner that recombinational repair occurs between them to form a new genome, and this new genome, upon replication, can initiate a new lineage.

Superinfection exclusion also occurs with phage T4. If a first-infecting phage T4 has no lethal damage in its genome, within one minute of metabolism, the first phage will block entry of a second phage T4 (superinfection exclusion) [23]. However, if the first phage T4 has lethal damage in its genome, such as from UV irradiation, the first phage will allow a superinfecting phage to enter, and genes of the first phage can then be rescued by recombination [24]. Phage T4 thus engages in sexual reproduction, which is of benefit when DNA damage is present in a first-infecting phage.

The mechanisms utilized by phage T4 during multiplicity reactivation could be deduced from an experiment utilizing UV-irradiated phages [25]. Two strains of phage T4 were UV-irradiated so that survival of the phage in bacterial host cells, where each bacterial cell was infected by a single phage, was at the level of 10^−3^. In one strain of phage T4, the genome carried eight conditional lethal mutations and the second phage strain had a genome that carried 24 conditional lethal mutations. A conditional lethal mutation means that the phage carrying that mutation could grow in a particular bacterial host called a permissive host, such as *Escherichia coli* CR63, but the mutation would be lethal in a restrictive host, such as *E. coli* S/6. Double infections were carried out on a permissive host. With UV irradiation (to cause DNA damage) followed by double-infection multiplicity reactivation, recombination frequencies between mutations increased about 4-fold. Thus, most progeny phages were recombinants. Infected permissive bacteria were diluted and placed in separate test tubes so that only a single infected bacterium was in each any test tube with an infected bacterium. When the progeny phage in a doubly infected bacterium completed the growth process, the bacterium would burst open and release all the progeny phage. Surprisingly, in 34 single bursts, 29 of the bursts had one or more progeny phage of the genotype of one or the other parent, with no recombination. We could interpret these findings to indicate that most progeny phages are the result of increased recombination, after irradiation, by a breakage and exchange mechanism. But also, some recombination, after irradiation, occurs by a copy-choice mechanism, leaving a highly marked parental genome without any recombination of markers.

Phage T4 has about 300 genes in its genome [26]. The phage T4 genome has all the 16 genes it needs for recombinational repair [27]. The repair of double-strand breaks, and other damages that require homologous recombinational repair, are carried out entirely by phage T4 gene products.

**Figure 4:**
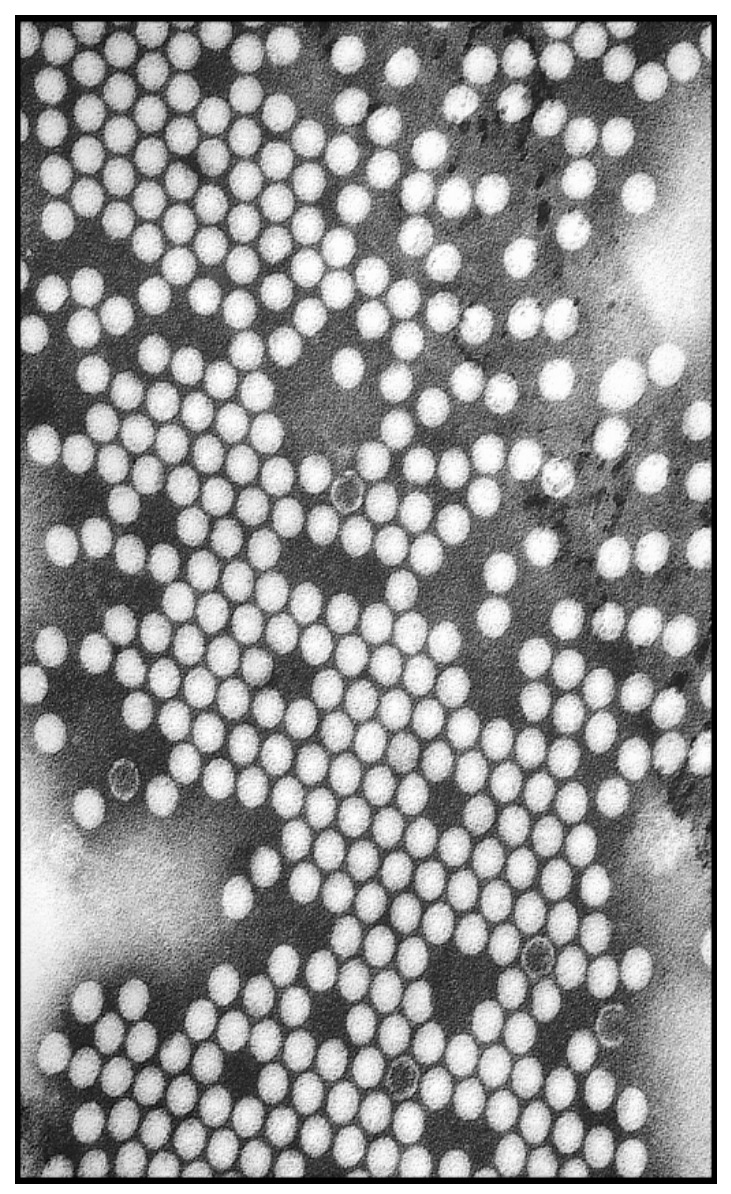
Poliovirus (electron micrograph) PD-USGov-HHS-CDC CDC/Dr. Fred Murray, Sylvia Whitfield.

In addition to bacteriophage, multiplicity reactivation has been found in a number of human pathogenic viruses, including herpes simplex, influenza, adenovirus, simian virus 40, vaccinia, reovirus, and polio (for poliovirus see Figure 4) [28]. The enhanced survival of these viruses due to multiplicity reactivation has been reported to occur when viruses undergo treatment with any one of several different DNA-damaging agents, including methyl methanesulfonate and N-Methyl-N′-nitro-N-nitrosoguanidine [29], trimethylpsoralen (which causes interstrand DNA cross-links) [30,31], and UV light [32]. In one of the experiments with herpes simplex [30], genetically marked genomes were used. Recombination was increased during multiplicity reactivation, indicating that recombinational repair was the basis for multiplicity reactivation.

In contrast to phage T4, multiplicity reactivation of herpes simplex virus appears to, in part, rely on the recombinational repair machinery of the host cell. This is shown because herpes simplex multiple infections of skin fibroblasts of Bloom’s syndrome patients have reduced multiplicity reactivation (Bloom’s syndrome patients are deficient in recombinational repair) [32].

Multiplicity reactivation in DNA viruses is a type of sporadic sexual reproduction. It utilizes recombinational repair and greatly increases survival when lethal damage is present in each of the genomes of multiply-infecting viruses.

## 4. Genetic Recombination in Bacteria: An Adaptation for DNA Repair

**Figure 5:**
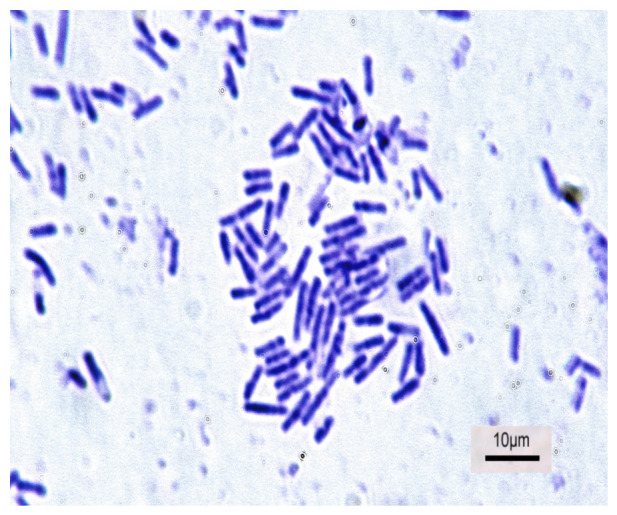
*B. subtilis* bacteria by Dr. Graham beards—own work, CC BY-SA 4.0.

Transformation in bacteria is a genetic recombination process. Bacterial DNA that has been released into growth media can be taken up by another bacterium of the same species and then integrated into the chromosome of the recipient cell. This process has the essential features of sexual reproduction, namely that genomes of separate parental origins interact and undergo recombination to produce a new genome. Transformation in bacteria depends upon the expression of many genes [33]. The DNA transfer process requires a recipient cell to adhere to the donor DNA suspended in the media, then take the DNA in, and finally undergo recombination to incorporate the donor DNA into its chromosome. To accomplish this, the recipient bacterium must change to a physiologic state called “competence.” For the bacterium *Bacillus subtilis* (see Figure 5) to enter competence, it needs to activate the expression of about 40 genes [34]. For *B. subtilis*, the transferred donor DNA can be greater in length than one million bases (1000 kb) and is likely double-stranded. Thus, the transferred DNA can be more than 1/3 the size of a whole chromosome, which is 4215 kb long [35]. We should also note, however, that although many bacterial species are restricted to recombination with the same species or closely related species, the *Agrobacterium* species complex is able to undergo transformation with widely divergent strains [36].

Many prokaryotes have the capability to carry out transformation, and Johnsborg et al. [37] listed 67 different prokaryote species, distributed among seven phyla, that can undergo transformation. Usually, cells undergo transformation to competence when cells are very crowded or there is a nutrient that is limiting. However, transformation to competence can also be caused by treatment with a DNA-damaging agent. As examples, competence and transformation can be induced in *Streptococcus pneumoniae* by mitomycin C (which causes DNA cross-links) or fluoroquinolone (which causes double-strand breaks by inhibiting topoisomerase) [38]. Experiments involving irradiating *B. subtilis* cells (see Figure 5) with UV light showed that transformation with donor DNA could repair recipient cell DNA damage [28,39,40]. DNA repair likely occurred through homologous recombinational repair. Bacterial recombinational repair depends on a RecA-type recombinase acting in homologous recombination [41].

Experiments with *Helicobacter pylori* showed that ciprofloxacin, an agent that causes DNA-double-strand breaks by interacting with DNA gyrase, causes the expression of competence genes, which then leads to an increased frequency of transformation [42]. Sixty-four different toxic molecules were tested for their ability to induce competence for transformation in *Legionella pneumophila* bacteria. Of these 64 toxins, only the six that caused DNA damage were able to induce competence [43]. Thus, in general, the induction of competence in bacteria appears to be an adaptive response to DNA damage. This response allows the interaction of a damaged chromosome with an exogenous chromosome to facilitate recombinational repair and restoration of an intact DNA genome sequence that, in turn, could be passed on to progeny.

Transformation in bacteria is a type of sexual reproduction. It utilizes recombinational repair and is able to increase survival when lethal damage is present in the recipient genome.

## 5. Meiosis and Recombination in Eukaryotes: An Adaptation for DNA Repair

In the contemporary double-stranded DNA world of eukaryotes, including animals, plants, fungi, and unicellular protists, the ability to sexually reproduce is widespread. Among animals and plants, only a very small fraction of species are considered to be asexual, with one in 100 being asexual for angiosperms and one in 1000 being asexual for animals [44].

Yeasts are eukaryotic fungi that have sporadic sexual reproduction, which primarily occurs under stress or unfavorable conditions, such as nutrient limitation. Sexual reproduction in these eukaryotes depends upon and requires the process of meiosis when generating progeny. During meiosis, the genomes that came from two parents become closely paired and undergo recombination. This recombination between non-sister chromatids of homologous chromosomes takes place during the pachytene stage of prophase I [45].

**Figure 6:**
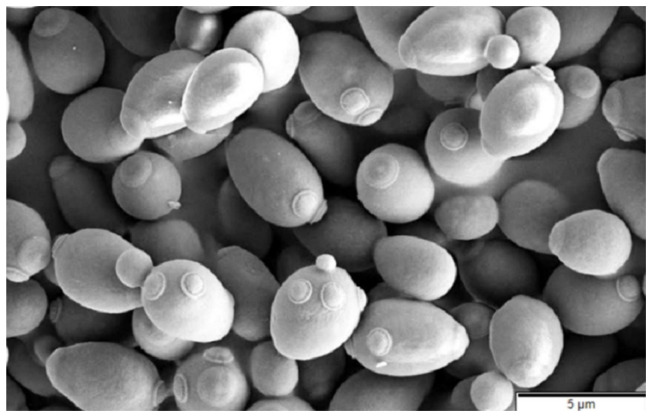
*S. cerevisiae* (electron micrograph) by Mogana Das Murtey and Patachamuthu Ramasamy CCBY 3.0.

The budding yeast *Saccharomyces cerevisiae* (see Figure 6 with buds on many cells) is a major model for meiosis research. Most genes that are central to meiosis in *S. cerevisiae* are also present in other eukaryotic sexually reproducing organisms [46]. Mitotic recombination occurs fairly infrequently in *S. cerevisiae* (about 10^−7^ to 10^−4^ per locus per generation). By comparison, meiotic recombination in *S. cerevisiae* occurs much more frequently (reaching 50% per locus per generation in some cases) [47].

**Figure 7:**
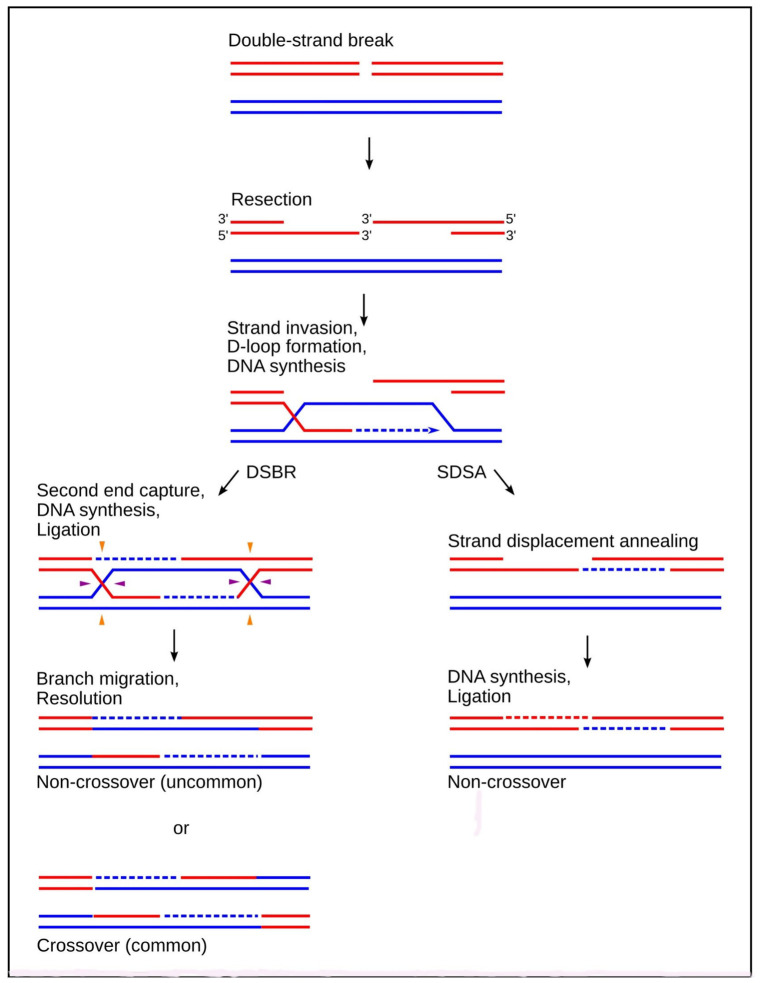
Two pathways of homologous recombination repair. The double-strand break repair (DSBR) pathway and the synthesis-dependent strand annealing (SDSA) pathway (a type of copy choice). By Em2012 CC BY-SA 3.0w.

During meiosis in *S. cerevisaie*, approximately two-thirds of recombination events occur by synthesis- dependent strand annealing (SDSA) [48]. This is a copy-choice type of recombinational repair (see Figure 7). SDSA is a DNA repair pathway that repairs a double-strand break without the exchange of chromosome arms. The SDSA repair of the double-strand break occurs by a localized replicative switch at the double-strand break from an initiating genome to a second genome and then a switch back to the initiating DNA duplex. In Figure 7, using a double-strand break as the lesion, SDSA is compared to the other major repair pathway, the double-strand break repair pathway (DSBR), which commonly results in the exchange of chromosome arms.

When *S. cerevisiae* finds itself in a good environment for growth, it enters a diploid state and replicates clonally by mitosis. On the other hand, if a nutrient is limited, this yeast undergoes meiosis, resulting in the formation of haploid spores [49]. The haploid spores then generate haploid cells that may then reproduce asexually by means of mitosis. In natural populations of *S. cerevisiae*, both clonal reproduction and selfing occur. Selfing consists of one cell mating with a sister cell arising from the same tetrad within a single ascus [50]. In naturally growing populations of *S. cerevisaie*, outcrossing occurs infrequently [51]. Using whole genome sequencing, it was found that outcrossing occurred only once in 50,000 cell cycles [51]. This indicates that genetic diversity, provided by outcrossing, is unlikely to provide the principal adaptive benefit of sexual reproduction in *S. cerevisiae* [52]. Instead, selfing must provide an immediate or short-term benefit, such as the repair of otherwise deleterious DNA damage, which is available through recombinational repair, during meiosis [53,54].

Another yeast switches from asexual reproduction to meiotic sexual reproduction, with recombinational repair, when it encounters an externally damaging situation. *Schizosaccharomyces pombe* is an ascomycete fungus, which is usually a haploid fission yeast. When *S. pombe* is subjected to hydrogen peroxide in growth media, causing oxidative DNA damage, there is a strong induction of sexual reproduction, evident by the induced occurrence of meiotic spores [55].

Thus, with yeasts as a model for eukaryotes, there is an indication that sexual reproduction, with frequent recombination during meiosis, is an adaptation for DNA repair.

## 6. The Parasexual Cycle in Some Fungi: An Adaptation for DNA Repair

Most fungi have sexual reproduction with meiosis [44]. However, some eukaryotic fungi have a parasexual cycle. These include, for instance, *Candida albicans* [56], *Aspergillus fumigatus* (which has both a meiotic sexual cycle and a parasexual cycle) [57], *Penicillium chrysogenum* [58], and *Fusarium oxysporum* [59]. The parasexual cycle starts with the fusion of cells, yielding cells with two separate nuclei. After two cells fuse, the nuclei then fuse, with the fused nucleus having double the original DNA content. During the multiplication of these cells, mitotic recombination occurs. Then, during continued multiplication, the nuclei lose chromosomes or chromosome segments until cells are formed with the original chromosomal DNA content.

*C. albicans* is well studied and can be used to illustrate the parasexual cycle. *C. albicans* is highly studied since it occurs on the skin, mouth, and gastrointestinal tract of about 70% of humans, mostly existing commensally, although it can also cause serious fungal infections [60].

*C. albicans*, growing as a commensal population, has diploid nuclei, usually within either a yeast-type form of single cells or as hyphae of connected cells. If *C. albicans* comes under oxidative stress (possibly from encountering host immune cells), the oxidative stress may cause DNA damage, including double-strand breaks [61]. *C. albicans* cells undergoing oxidative stress may develop mating projections on the cells or hyphae, allowing cells or hyphae to mate [62], forming tetraploid cells [63]. The tetraploid cells have high levels of recombination, comparable to the levels of recombination in cells with meiosis [56]. The recombination in *C. albicans* is by homologous recombination (not by non-homologous end joining), and serves to repair DNA damage due to UV light or methane-methyl-sulfonate (MMS) [64].

Thus, in parasexual reproduction in C. albicans, homologous recombinational repair is able to increase survival when lethal damage due to UV light or MMS is present in the genome.

### Sexual Reproduction with Meiosis in Protozoa: An Adaptation for DNA Repair

**Figure 8:**
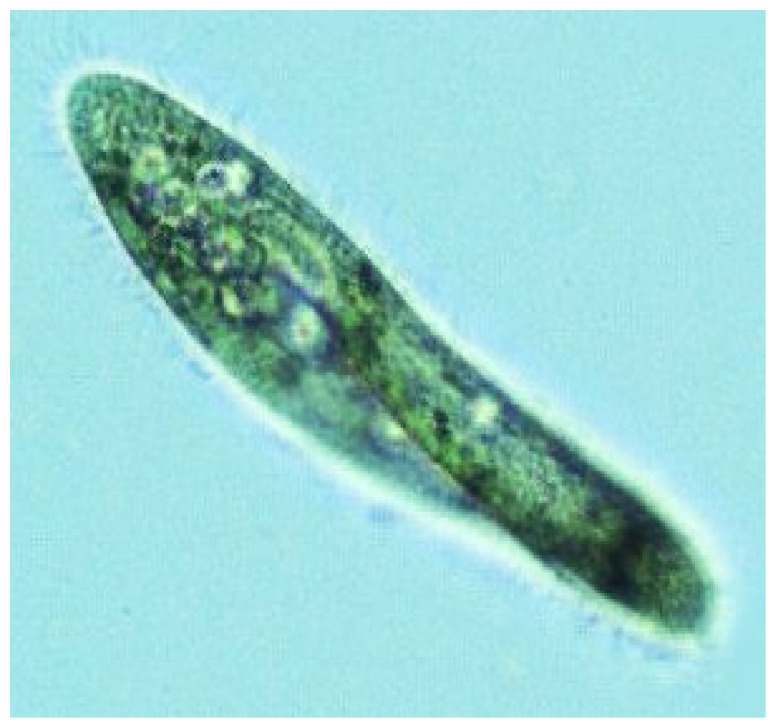
*Paramecium tetraurelia* by DavidpBowman CC BY-SA 4.0.

*Paramecium tetraurelia* (see Figure 8) is a well studied protozoan. *P. tetraurelia*, when replicating asexually, has a transcriptionally silent pair of diploid micronuclei that provide the germ-line DNA, and a very large and transcriptionally active macronucleus. The macronuclear DNA content of *P. tetraurelia* is about 2.42 × 10^11^ base pairs, and the haploid DNA of the micronucleus is about 2.9 × 10^8^ base pairs, giving a macronuclear-to-haploid micronucleus DNA ratio of about 800-1 [65].

When *Paramecia* undergo sporadic sexual reproduction (conjugation) or self-fertilization (autogamy or automixis), the macronucleus becomes fragmented, slowly shrinks, and is absorbed into the cytoplasm. The cells then have a pair of micronuclei. During autogamy, the micronuclei undergo meiosis, producing eight haploid micronuclei. Seven of these disintegrate. The single surviving haploid nucleus then undergoes a mitotic division to produce two identical haploid gamete nuclei. These two nuclei fuse to produce a diploid nucleus. This diploid nucleus mitotically divides twice to produce four micronuclei. Two micromuclei remain as a pair of germline micronuclei, and two begin to form a macronucleus. Macronuclear formation involves making copies of the two non-germline micronuclei with rearrangements of the germline genome. This involves fragmentation of micronuclear chromosomes, which then obtain telomere additions to form the macronuclear chromosome ends, as well as the deletion of internal eliminated sequences. This is followed by extensive replication to form the macronucleus [66].

During asexual reproduction (binary fission) in *Paramecium*, the large macronucleus divides by a simple process called amitosis, while the smaller micronuclei undergo true mitosis, with the cell then splitting transversely. Amitosis involves simple splitting of the DNA content without spindle formation.

In asexually dividing lines, the clone that is formed shows increasing signs of aging, shown by a reduced rate of cell fission and a reduced number of food vacuoles per cell for digesting food [67]. In *Paramecium*, the asexual clone of cells all lose vitality, and the clone dies out after roughly 200 cellular fissions unless cells of the clone undergo a meiotic process, either autogamy or conjugation.

Functional alterations in the macronucleus, rather than the cytoplasm, of paramecia were shown to be responsible for clonal aging [68]. During clonal aging, DNA damage, as shown by increased single-strand breaks in DNA, increases dramatically [69,70,71].

When clonally aged *P. tetraurelia* undergo meiosis, which can occur during either conjugation or automixis, the progeny produced are rejuvenated. The progeny become capable of undergoing many further mitotic binary fission divisions. There is very little DNA damage in the newly formed macronucleus.

Meiosis in *P. tetraurelia* is quite clearly an adaptation for DNA repair and rejuvenation [72]. The CtlP protein of *P. tetraurelia* is crucial for the structural nuclear changes in meiosis during sexual reproduction and needed for the production of viable progeny [72]. The nuclease protein complex containing CtlP and Mre11 is needed for the recombinational repair of double-strand breaks during meiosis [72].

Under starvation conditions, *P. tetraurelia* can undergo meiosis and self-fertilization, and the benefit of self-fertilization is not at all dependent on any new genetic variation in progeny [73]. These findings suggest that meiosis provides a fitness advantage related to DNA recombinational repair that is independent of any accompanying effect of sex on genomic diversity [73,74].

Thus, in a protzoan, it seems that clonal aging is caused by progressive additions of breaks in the DNA of the macronucleus, and that reversing aging (rejuvenation) depends on recombinational repair of germline DNA (the micronuclei) during meiosis.

## 7. Genetic Recombination in Insects: An Adaptation for DNA Repair

**Figure 9:**
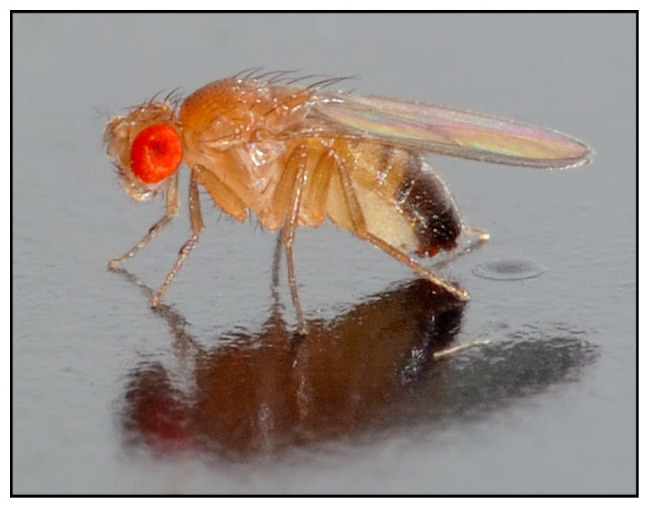
*D. melanogaster* by Andre Karwath CC BY-SA 2.0.

Most insect species have obligate sexual reproduction, although aphids and bees, and some other insects, utilize both sexual and asexual reproduction, having sporadic sexual reproduction. Meiosis occurs in almost all insect sexual reproductive systems, and appears to have the same benefit as within other eukaryotes. *Drosophila melanogaster* (see Figure 9) is another model organism for studies of the adaptive function of meiosis, particularly at the molecular genetic level. Meiotic recombination in *D. melanogaster* is increased by exposure to the DNA-damaging agents ultraviolet light [75] and mitomycin C [76], indicating that genetic recombination is employed in repairing damage in germline DNA.

The *D. melanogaster* genes mei9 and mei41 code for enzymes needed in meiosis. Mutants with defects in these two genes have both decreased meiotic recombination as well as hypersensitivity to agents that damage DNA, including UV, X-rays, methyl methansulfonate, as well as the DNA-alkylating agent nitrogen mustard [77]. The single mutations causing alterations in both meiotic recombination and repair of damage to DNA indicate that meiotic recombination has a role in the repair of DNA damage during meiosis.

## 8. Genetic Recombination in Outcrossing Plants: An Adaptation for DNA Repair

Plants may reproduce by an outcrossing sexual meiotic process, a parthenogenetic meiotic process, or by a vegetative reproductive process that does not involve meiosis.

Outcrossing sexual reproduction ordinarily involves the production of haploid gametes by meiosis followed by fertilization.

Parthenogenic meiotic processes include apomixis, apomictic parthenogenesis, automixis, and selfing. Fertilization is the union of gametes from separate lineages to produce a new progeny diploid lineage. In plants, meiosis provides an effective recombinational DNA repair capability for dealing with certain types of DNA damage. This includes repairing some oxidative DNA damage in germline reproductive tissue [78]. During meiosis in the plant germline, recombinational repair ameliorates the double-strand breaks that are produced in the DNA genome. This recombination process employs the proteins coded for by the genes DMC1 and RAD51. These proteins are homologues of DNA recombinases that eukaryotes employ in general [79]. Central features of eukaryotic meiosis usually consist, first, of the pairing of homologous chromosomes. Then, double-strand breaks are formed, followed by recombinational repair [80]. Such processes seem to be adapted for effective repair of at least some types of DNA damage in the germline [80].

**Figure 10:**
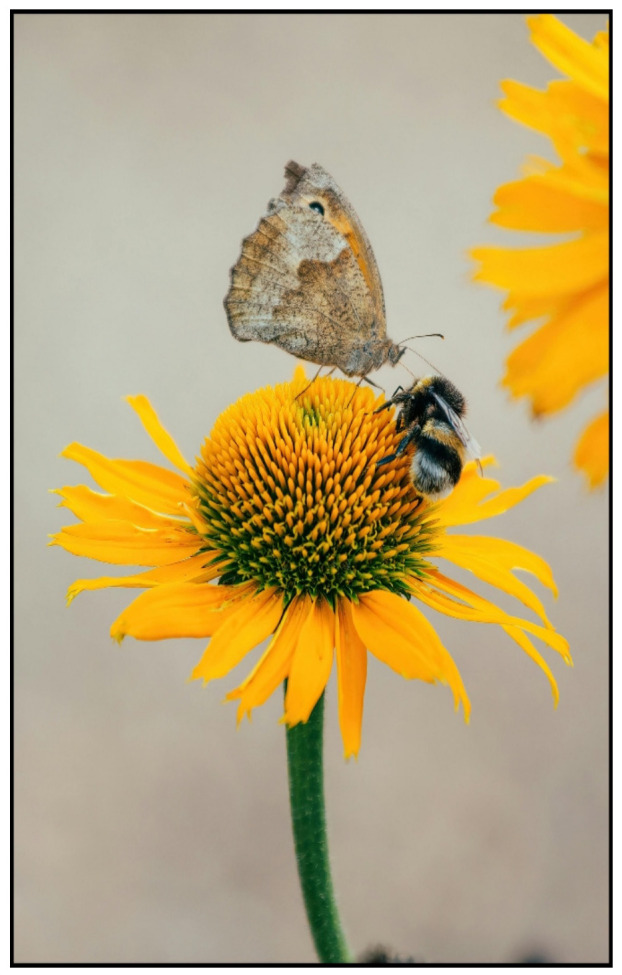
A cone flower with bee and butterfly by Konin, Wojewodztwo wielko-polskie, Polska In the Public Domain.

In flowering plants (see Figure 10), there appear to be two fundamental aspects of outcrossing sexual reproduction. The first is meiosis, which is maintained by the advantages of the repair of germline DNA. The second is cross-fertilization (outcrossing) that is maintained by the advantages of complementation (the masking of recessive deleterious alleles) [74].

The facultatively sexual, multicellular green alga *Volvox carteri* is able to undergo sexual reproduction upon induction by oxidative stress [81]. The genes of *V. carteri* required for sexual reproduction are activated by relatively higher levels of cellular unstable, oxygen-containing molecules that easily react with other molecules in a cell (likely to cause DNA damage) [82]. The induction of sex in *V. carteri* can be inhibited by exposure to antioxidants [82,83]. On the basis of these findings, it was proposed that in the early evolution of *V. carteri*, sexual reproduction emerged as a possible response to DNA damage caused by reactive oxygen species (ROS) [82]. Damage to DNA caused by ROS may be repaired while undergoing the process of meiosis that occurs during the germination of the zygospore that gives rise to a new generation [83].

## 9. Meiosis and “Asexual” Modes of Reproduction in Plants

The great majority of “asexual” types of reproduction in plants retain meiosis, but often in a somewhat altered form or else as a possible alternative pathway [84]. Mirzaghaden and Hörandl [80] reviewed the origin of and the mechanisms employed in normal meiosis as well as in the alternate forms of meiosis that are utilized by uniparental (non-sexual or non-mating) reproduction. For instance, they point out that apomixis (the generation of progeny without fertilization by males) in ferns involves a pre-meiotic doubling of chromosomes in a somatic cell followed by a meiosis that produces diploid spores. This retains the benefits of recombinational repair during meiosis, but without mating. Reviewing a number of types of asexual reproduction [80], they concluded that these asexual forms of reproduction usually retained steps including pairing of homologues, the formation of double-strand breaks, and the recombinational repair at prophase I as the most indispensable steps in forming progeny. They point out that these retained steps in uniparental reproduction appear to be more adapted for repairing oxidatively damaged DNA rather than for generating genetic diversification [80].

## 10. Repair of Mammalian Oocyte DNA by Recombination During Meiosis

At birth, female mammals and female birds already have all the oocytes needed for ovulation during their lifespan [85]. During adult development, their oocytes remain in the prophase I stage of meiosis. At this stage, they have two sister chromatids of each chromosome, which constitute four copies of each chromosome (called the 4C stage of meiosis) [85]. This arrest at the 4C stage allows recombinational repair of DNA damage between sister- as well as non-sister homologous sequences.

**Figure 11:**
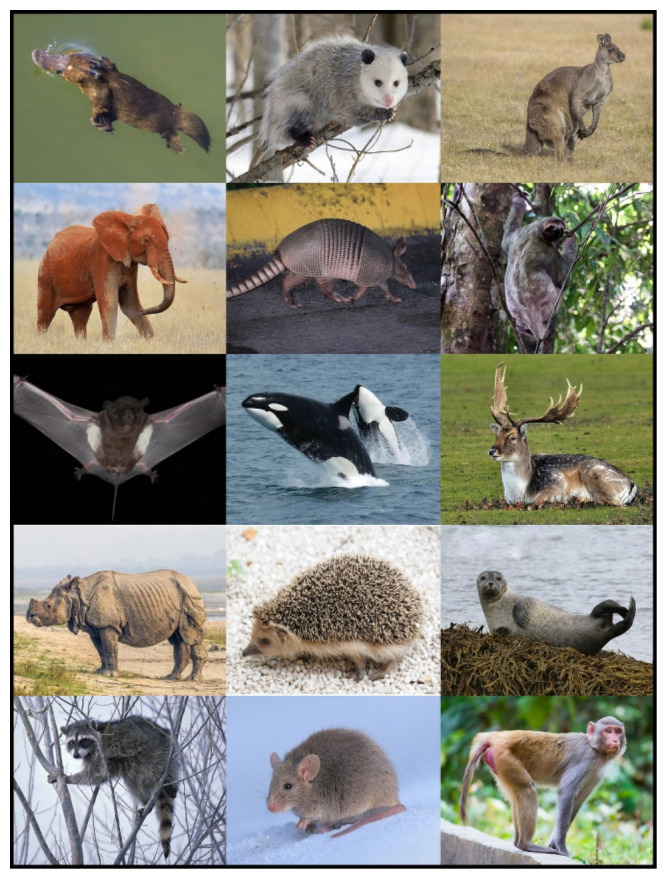
Mammals. By ZkevinTheCat CC BY 4.0.

Mammals (see Figure 11) are often very long-lived so that their oocytes tend to accumulate DNA damage and epigenetic alteration during the female’s lifetime [86]. During the maturation of oocytes, some of the types of damage in the DNA of oocytes are repaired by the processes of breakage and exchange recombinational repair or by a process that fuses broken ends together, without requiring a homologous chromosome [86]. Breakage and exchange recombinational repair utilizes the exchange of undamaged genetic sequence information from a chromosome to correct the damaged portion of its homologous partner chromosome. The numerous DNA recombinational repair proteins available during the maturation of an oocyte are actively recruited to the locations of some types of damaged DNA. Polymerase delta, in particular, is critical for oocyte DNA synthesis in the breakage and exchange recombinational repair of these DNA damages [86]. DNA double-strand breaks in oocytes, in particular, can be repaired by a process involving RAD51-mediated homologous recombination [87].

## 11. Repair of Mammalian Spermatocyte DNA During Meiosis

Spermatocytes are able to mend double-strand DNA breaks and other types of damaged DNA when spermatocytes have entered the prophase stage of meiosis. Damage to DNA is often caused by oxidative free radicals created by mitochondria during normal metabolism. In spermatocytogenesis, special DNA repair processes are utilized during meiosis so that the appropriate DNA sequence is passed on to progeny [88]. These repair processes include homologous recombinational repair and non-homologous end joining [88].

For mice, recombinational repair utilizing homologous chromosomes that mend double-strand breaks happens during the different stages of spermatogenesis [89]. Within mouse spermatocytes, homologous recombinational repair is mostly seen to occur during the pachytene stage of meiosis. During this stage, a copy-choice mode of homologous recombinational repair, resulting in gene conversion, predominates. Within other stages of spermatogenesis, however, a breakage and exchange mode of homologous recombination appears to be more common [89]. In the process of mouse spermatogenesis, mutations within genomes of cells at various stages, including pachytene spermatocytes, occur at approximately 5 to 10 times lower frequencies than frequencies of mutations seen within somatic cells [90]. The elevated repair ability of spermatocytes appears to have a major role in obtaining such reduced mutation frequencies, thus maintaining the genomic integrity of mouse male germlines.

## 12. Advantage of Outcrossing

The main focus of this presentation has been that sexual reproduction arose early in evolution and has been maintained as a means of dealing with genetic damage. However, it is also important to note that in many species, outcrossing sexual reproduction provides an additional advantage related to the ability to mask the expression of recessive deleterious alleles during the diploid phase of the life cycle. Such a masking effect is called genetic complementation. The masking can also be referred to by the terms hybrid vigor, heterosis, and heterozygote advantage. In species in which outcrossing has been the principal mode of reproduction, circumstances that limit outcrossing while allowing inbreeding may have deleterious consequences. This phenomenon, referred to as inbreeding depression, is thought to be largely due to the expression of recessive deleterious mutations [91]. Many species have evolved mechanisms to avoid inbreeding, as indicated by the following examples.

Experiments in mice involving in vitro fertilization indicated the occurrence of sperm selection at the gametic level [92]. It was observed that when the sperm of brother and non-brother males were combined, the sperm of the non-brother males was more successful in fertilization compared to the sperm of the brother males. These findings indicated that eggs may have a bias against related sperm.

In competition trials, male fruit flies (*Drosophila meanogaster*) of four different degrees of genetic relatedness were mated with female fruit flies [93]. There was a bias against sperm that came from males more closely related to the female fruit flies.

There is a small population of highly inbred grey wolves (*Canis lupus*) that live on an island: Isle Royale National Park, MI, USA. These wolves are exhibiting population decline and are almost extinct because of the presence of homozygous recessive deleterious mutations leading to a decrease in viability [94,95].

Avoidance of inbreeding is very variable among animals [96]. Avoidance of inbreeding through mate selection appears to primarily evolve under circumstances where there is risk of inbreeding depression combined with frequent encounters between potential sexual partners that are genetically related to each other [96].

## 13. Meiosis Associated with Parthenogenesis in the Animal Kingdom

Although outcrossing sexual reproduction is the principal means of reproduction in most eukaryotic species, reproduction involving meiosis without outcrossing also occurs.

Rotifers are microscopic animals of the class Bdelloidea that have existed for a long time without outcrossing [97]. The longevity of the asexual bdelloid rotifer clade is greater than 60 million years [98]. Homologous recombination appears to occur either during a modified meiosis (when resolving programmed DNA breaks) or during mitotic DNA double-strand break repair [98]. In the bdelloid species *Adineta vaga*, a noncanonical meiosis seems to be the means employed in germline DNA repair [97].

Facultative parthenogenesis occurs in many organisms of the animal kingdom [99]. As an example, king cobra snakes are able to undergo facultative parthenogenesis [99]. Parthenogenesis in this case occurs by a modified type of meiosis called “terminal fusion automixis.” In this process, the meiotic products produced at anaphase then fuse together [99].

**Figure 12:**
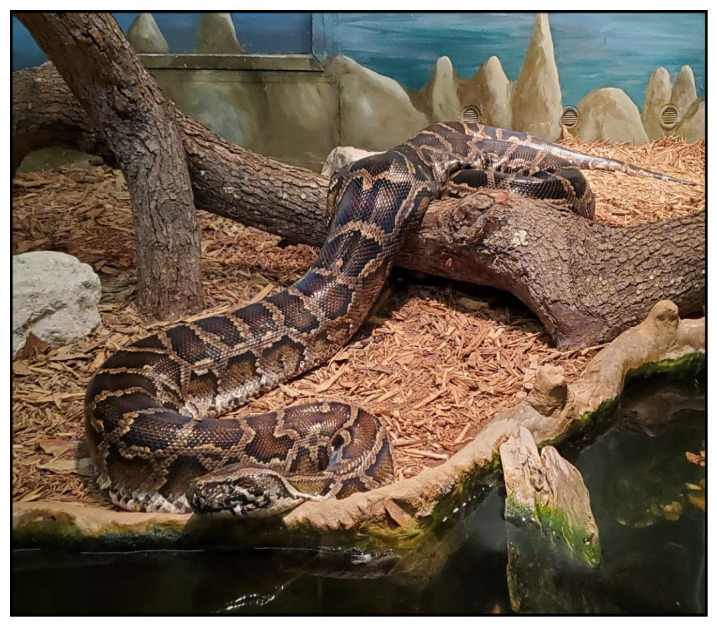
Burmese python. JJonah Jackalope CC BY-SA 4.0.

The Burmese python (see Figure 12), when maintained in captivity, was demonstrated to be able to reproduce asexually [100]. Offspring are clones of their mother, and the reproductive process is a form of parthenogenesis involving a variation in meiosis [100].

Female Ambystoma unisexual mole salamanders are present in the North American Great Lakes region [101]. These unisexual salamanders are the most ancient known unisexual vertebrates. They emerged as a species about 5 million years ago [102]. They engage in kleptogenesis. Females take sperm from males of a bisexual species that live in the same area. The acquired sperm serve to stimulate meiosis but do not contribute genetically to offspring [103].

These examples illustrate that although sexual reproduction, involving both the meiotic production of gametes and outcrossing, is the most usual mode of producing offspring in the animal kingdom, reproduction by a parthenogenetic meiotic process also can occur. Under certain circumstances, particularly when there are substantial costs in finding mating opportunities, a parthenogenetic strategy involving meiosis may be the most effective means of reproduction. In these cases, meiosis retains the ability to carry out recombinational repair of damage to DNA in the germline, and it occurs without introducing new genetic variation.

## 14. Small but Clear Adaptive Advantage of Genetic Variation Due to Recombination During Sexual Reproduction

As reviewed by Sarah P. Otto [104], there are more costs to sexual reproduction than to for an organism with asexual reproduction. There is an initial two-fold cost since a female or male parent may transmit only half of their genes to the next individual progeny member, compared with all of their genes for an asexual parent. In addition, recombination in sexual reproduction may break apart good combinations of genes that were present in a successful parent. Further, mitotic asexual reproduction may take much less time than that needed for complicated meiosis during sexual reproduction. Otto asks, “Given the costs of sex and the widespread potential for asexual reproduction, why do so many species reproduce sexually?” Many biologists have proposed that sex and recombination evolved because they give rise to the variation needed by selection. This was first proposed by August Weisman in 1889.

**Figure 13:**
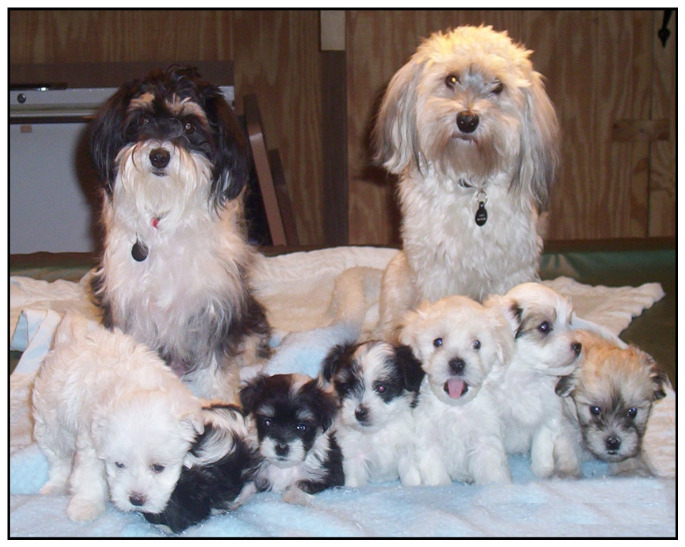
Litter of Havanese puppies. By Cartman0052007 CC BY 3.0.

The variation produced by sexual reproduction is exemplified in the image shown in Figure 13. In this Figure, the puppies produced by a single mating have varied coat colors and varied sizes.

However, Otto [104] describes the many unsuccessful attempts to mathematically find a benefit in the variation produced by recombination during sexual reproduction that exceeds the costs of sex, and explains the difficulties with each model so far.

The major selective value of sexual reproduction appears to be the repair of otherwise lethal damage within a genome that is passed from a parent to progeny [78,105]. In Figure 2, one can see that damage that is lethal in single infections with one genome present is no longer lethal when recombinational repair can occur in multi-infected cells. The need for survival (repair of otherwise lethal genome damage) can account for the costs of sexual reproduction.

However, in a varied environment, the different recombinant progeny from sexual reproduction can produce individuals with different properties, where a particular recombinant would have greater adaptive ability in a niche of that environment. This would be especially true, for instance, in the case of *Candida albicans*, described above. *C. albicans* is present in the different environments of the skin, mouth, and gastrointestinal tract, plus in infective sites. In each of these areas, the various recombinants of parasexual recombination would compete, and a particular recombinant that is well adapted to that particular area could have a great advantage and could rapidly spread asexually. This illustrates a particular adaptive advantage to sexual reproduction in addition to recombinational repair.

Another advantage of sexual reproduction, in addition to recombinational repair, is evident in an environment where parasites can be a problem. As an example, Auld et al. [106] showed that there was strong *Pasteuria ramosa* parasite adaptation with the ability to grow within an asexual group of *Daphnia magna*, but the parasite had much more difficulty growing in varied sexual offspring of *D. magna*.

A third added advantage of sexual reproduction is its advantage in speeding up evolutionary progress for a population. This is because sexual reproduction with recombination can allow good mutations arising in separate mating individuals to recombine in a single progeny individual. The single progeny individual could then reproduce and spread the good combination. This was demonstrated in parallel *E*. *coli* populations, where one set of populations was recombination-positive, and the other set of populations was recombination-negative [107].

## 15. Discussion

Genetic recombination, a central feature of meiosis in eukaryotes, is likely kept as a common part of the reproductive process because it promotes recombinational repair of damage within the DNA in the germline. [74]. The association of outcrossing with meiosis is likely maintained because outcrossing promotes the covering up of recessive deleterious mutations within progeny (hybrid vigor through complementation) [74].

Among organisms that have high levels of outcrossing, the genetic variation that is produced by a combination of recombinational repair and complementation may have a positive selective value [74]. However, it has also been argued that producing more variable offspring is not necessarily favorable [108]. In addition, outcrossing may be absent as in obligate parthenogens with meiotic capability. Or outcrossing may be limited if the population is very dispersed so that finding a mate is costly. The production of genetic variation may sometimes be a beneficial outcome of sexual reproduction. However, the information reviewed here indicates that the primary benefit of the origin and continuing evolution of sexual reproduction has been recombinational repair or avoidance of genome damage.

## Data Availability

No new data were created or analyzed in this study. Data sharing is not applicable to this article.
